# Distinct Protein Classes in Human Red Cell Proteome Revealed by Similarity of Phylogenetic Profiles

**DOI:** 10.1371/journal.pone.0054471

**Published:** 2013-01-21

**Authors:** Paweł Szczesny, Agnieszka Mykowiecka, Krzysztof Pawłowski, Marcin Grynberg

**Affiliations:** 1 Institute of Biochemistry and Biophysics, Polish Academy of Sciences, Warsaw, Poland; 2 Department of Plant Molecular Biology, Institute of Experimental Plant Biology, University of Warsaw, Warsaw, Poland; 3 University of Warsaw, Warsaw, Poland; 4 Faculty of Agriculture and Biology, Warsaw University of Life Sciences, Warsaw, Poland; 5 Nencki Institute of Experimental Biology, Polish Academy of Sciences, Warsaw, Poland; Institute of Molecular Genetics IMG-CNR, Italy

## Abstract

The minimal set of proteins necessary to maintain a vertebrate cell forms an interesting core of cellular machinery. The known proteome of human red blood cell consists of about 1400 proteins. We treated this protein complement of one of the simplest human cells as a model and asked the questions on its function and origins. The proteome was mapped onto phylogenetic profiles, i.e. vectors of species possessing homologues of human proteins. A novel clustering approach was devised, utilising similarity in the phylogenetic spread of homologues as distance measure. The clustering based on phylogenetic profiles yielded several distinct protein classes differing in phylogenetic taxonomic spread, presumed evolutionary history and functional properties. Notably, small clusters of proteins common to vertebrates or Metazoa and other multicellular eukaryotes involve biological functions specific to multicellular organisms, such as apoptosis or cell-cell signaling, respectively. Also, a eukaryote-specific cluster is identified, featuring GTP-ase signalling and ubiquitination. Another cluster, made up of proteins found in most organisms, including bacteria and archaea, involves basic molecular functions such as oxidation-reduction and glycolysis. Approximately one third of erythrocyte proteins do not fall in any of the clusters, reflecting the complexity of protein evolution in comparison to our simple model. Basically, the clustering obtained divides the proteome into old and new parts, the former originating from bacterial ancestors, the latter from inventions within multicellular eukaryotes. Thus, the model human cell proteome appears to be made up of protein sets distinct in their history and biological roles. The current work shows that phylogenetic profiles concept allows protein clustering in a way relevant both to biological function and evolutionary history.

## Introduction

Human blood is composed of cells and plasma. The most abundant cell is the erythrocyte, which lacks the nucleus in mammals, as opposed to all other vertebrates. Red blood cells are oval, red due to hemoglobin which occupies most of the cell (98% of the overall cytoplasmic protein content) and flexible to move freely through capillary vessels [1]. Specialization of the erythrocyte is visible in its extraordinary structure. It is shaped like a biconcave disc and originates in the bone marrow where reticulocytes, first developmental stages of red blood cells, are produced. Until the end of its life span of 120 days, with 120 miles of travel and 1.7 10^5^ circulatory cycles, the human RBC has successfully coped with a number of dangers, such as passages across narrow capillaries and splenic slits, periodic high turbulences and high shear stresses, along with extremely hypertonic conditions [2]. The main function of erythrocytes is to transport oxygen and carbon dioxide. Additionally, these cells have been shown to play several key roles, like calcium and redox homeostasis [3], [4], cell proliferation [5], immunity [6], ROS production [7] and sugar transport [8]. Other vital roles of erythrocytes may be expected.

All these functions are related to proteins functional in a mature cell. In recent works, authors have identified around 1900 erythrocyte proteins [9]. Except for hemoglobin, the proteins making up remaining 2% of the erythrocyte proteome mass are involved in a plethora of functions in which protection of accumulated protein machinery from oxidative stresses seems to be central to preserve its life [9], [10]. Recent work by D'Alessandro and colleagues identifies about 50 molecular networks in red blood cells that in majority belong to the following categories: molecular transport, protein synthesis, cellular assembly and organization, post-translational modification and protein folding, cellular function and maintenance, protein degradation and cell death [9]. Here, we present an analysis in a somehow similar spirit, albeit dissecting the proteome along evolutionary lines.

The phylogenetic profile concept has been introduced by Eisenberg and colleagues more than twenty years ago [11]. Basically, similar patterns of presence and absence of protein families in various organisms were shown to correspond to similar functions. Phylogenetic profiles have been employed in a number of studies, however, they were always used as an aid in further characterization of proteins, e.g. predicting protein-protein interactions and functional relationships such as in the PROLINKS and STRING servers [12]–[14]. Additionally, very recently a software package, ProPhylo, has been released designed to perform phylogenetic profiling studies [15]. ProPhylo addresses the major issue in such studies, that is reliably achieving granularities in similarity matrix required to obtain representative protein clusters. Here, we approached the problem from a different angle. We used the Fruchterman-Reingold graph-layout algorithm combined with a simple scoring system to identify evolutionary similarities in human red blood cell proteome.

In our work, we combine the proteomic and functional data with the evolutionary history of the erythrocyte proteome. Such a combination of diverse data sources allowed us to identify “young” and “old” sets of proteins in the red blood cell, which automatically separates “old” from “new” pathways in erythrocytes.

## Materials and Methods

### Data sources

We have used a previously published comprehensive set of erythrocyte proteins as a starting point [9]. This set was complemented with high-abundance erythrocyte proteins as listed in [16]. While D'Alessandro and colleagues [9] originally report over 1900 entities, we have found that less than 1500 gene identifiers could be reliably mapped to identifiers of Universal Protein Resource (UNIPROT) at the time of conducting this study. This discrepancy is partially a result of dynamics in biological data repositories – databases used by us in the course of this study were more recent than D'Alessandro and co-workers had used. Additionally, a large number of entities in the above study referred to “groups” or “complexes” and as such were simply redundant.

### Construction of phylogenetic profiles

For each of 1410 human erythrocyte proteins, three iterations of PSI-BLAST sequence search [17] were performed on the Refseq Protein database (version from March 2012), using E-value threshold of 0.001. Hits to protein sequences longer than 120% of the query sequence, and those shorter than 80% of the query sequence were discarded. Then, for each query protein, the phylogenetic profile was constructed: a vector L indexed by identifiers of selected species from the NCBI Taxonomy database.

Alternative approach that used orthologous groups relied on information provided by eggNOG 2.0 database [18]. Its authors provided a mapper between Uniprot identifiers and orthology groups.

### Calculation of phylogenetic profile similarity

For two phylogenetic profiles, L_i_ and L_j_, a scalar product D_ji_ was calculated L_i_ * L_j_ equal to the number of species common to the two profiles. For two proteins, *i* and *j*, we define the similarity S(i, j) of their two profiles as follows.
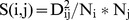
where N denotes length of vector L of the given phylogenetic profile.

### Python implementation of the algorithm

The algorithm together with necessary scripts to automate PSI-BLAST searches was implemented using the Python package. The source code and documentation are available at http://github.com/agnmyk/simphypro. It is worth noting that bitwise operations are preformed using Python bitarray package. In comparison to the solution where Python dictionaries are used to represent information on taxon ids, utilizing bitwise operations resulted in a substantial gain in efficiency. For a test set of 13000 proteins the processing time was reduced about ten times.

### Clustering of proteins using phylogenetic profile similarity

Similarity S was taken into account only for pairs of proteins for which shorter of phylogenetic vectors had a length of at least 60% of the longer one. This condition was introduced to avoid a situation in which natural connections between the majority of proteins (coming from the fact they almost all are present in Metazoa) would result in lowering the resolution of the clustering.

The final analysis was performed using the CLANS software [19]. The proteins were clustered using Fruchterman-Reingold graph-layout algorithm. The picture of clusters was obtained using similarity threshold of 0.6 for clarity, however the clusters were defined using all pairwise similarities meeting the 60% length requirement (see above). We used convex clustering method of CLANS software (which groups all sequences with average sequence-to-cluster attractive forces better than 0.5 of standard deviation of the average attraction for the dataset) to identify final clusters.

### Cluster analysis

The functional analysis of clusters was performed using the David systems biology tools [20]. For the statistical analysis of functional term enrichment, the full erythrocyte protein set was used as background. Several annotation sources were used, e, g, Gene Ontology, SwissProt, KEGG, InterPro, Pfam. Thus, functional annotations related to biological processes and molecular functions were considered together in the same manner. The figure showing counts of erythrocyte proteins mapped onto taxonomic tree was obtained with iTOL service [21]. Venn diagram was obtained with VENNY tool [22].

## Results

### Clustering of the HRBC proteome by similarity of phylogenetic profiles

**Figure 1:**
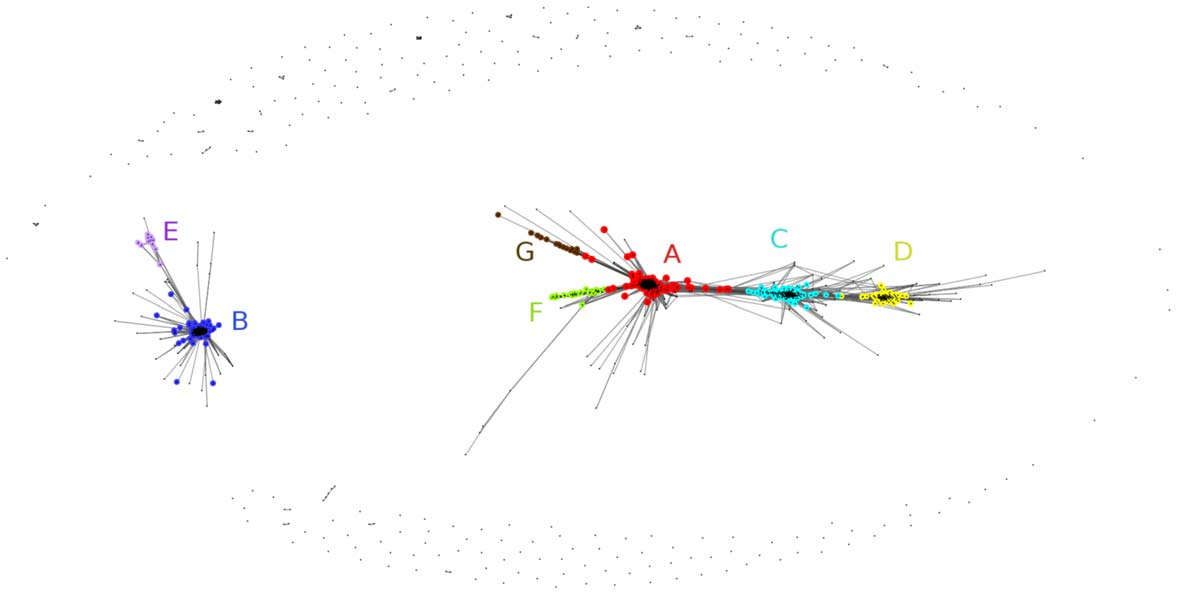
Phylogenetic clustering of erythrocyte proteins. Red: cluster A, dark blue: cluster B, light blue: cluster C, yellow: cluster D, magenta: cluster E, green: cluster F, brown: cluster G, grey: unclustered cloud. Plot in log scale.

Out of 1410 human erythrocyte proteins, 1409 had at least one significant hit to a non-human protein in a BLAST sequence similarity search. Using phylogenetic profile similarity threshold equal to 0.6 (see Methods), a picture with seven distinct clusters and an unclustered “cloud” emerges (see Fig. 1). The seven clusters group in two “super-clusters”. Additionally, there are a few proteins that tend to connect clusters (the effect is more pronounced when lower threshold of similarity score is used), however as the connections appear in the same order as the increase in average length of taxonomic hit vector per cluster, we consider them artifacts of the method. Summary of the clusters content (number of proteins and overview of taxonomic distribution) is presented in Table 1.

**Table 1:** Summary data for obtained clusters.

Cluster ID	No of proteins	Percentage of HRBC proteome	Overview of taxonomic distribution
**A**	521	37%	Mostly Eukaryotes
**B**	210	15%	Bacteria, Archaea and Eukaryotes
**C**	176	12%	Mostly *Metazoa*
**D**	78	6%	Mostly *Chordata*
**E**	11	<1%	Like B, but with some branches missing from all three classes
**F**	29	2%	Eukaryota and Archaea
**G**	15	1%	Like B, but with some branches missing from Bacteria
**CLOUD**	370	26%	All three classes but with the strongest representation of *Metazoa*

For detailed breakdown of taxonomic distribution at the phylum level, see Fig. 4.

**Figure 2:**
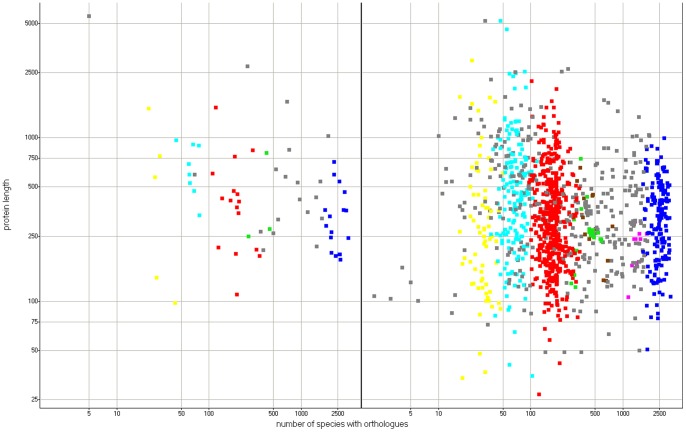
Protein length versus no of taxonomy hits. Left pane – disease mutation proteins. Right pane – other proteins. Red: cluster A, dark blue: cluster B, light blue: cluster C, yellow: cluster D, magenta: cluster E, green: cluster F, brown: cluster G, grey: unclustered cloud. Plot in log scale.

Overall, there is no apparent relationship between the number of taxonomy hits (number of species possessing a homologue) and query protein length (see Fig. 2), both for proteins known to harbour disease mutations (see Methods) and for all other proteins, yet a fraction of the longest proteins tend to have fewer taxonomy hits. This is not surprising since long, multidomain proteins tend to occur more often in fewer organisms – mostly in the multicellular eukaryotes [23], [24]. When individual clusters are examined, they do differ in average protein length (ANOVA P-value below 0.0001). The cluster C, with average protein length above 600 residues, differs significantly in average protein length from the other clusters. Also, the unclustered cloud (avg length above 500 residues) differs significantly from clusters A, B, C, E and F).

**Figure 3:**
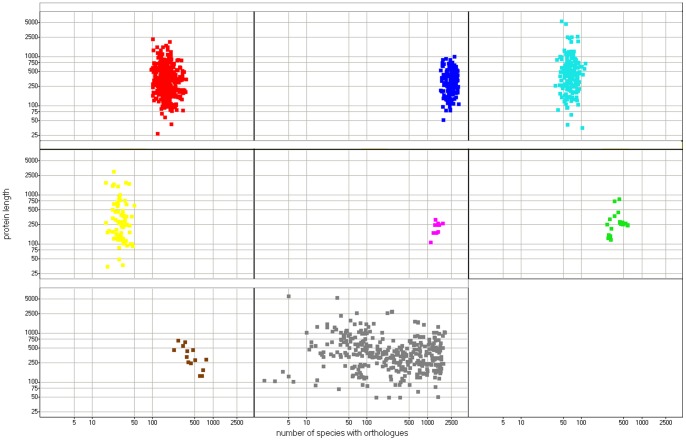
Protein length versus number of taxonomy hits, by cluster. Red: cluster A, dark blue: cluster B, light blue: cluster C, yellow: cluster D, magenta: cluster E, green: cluster F, brown: cluster G, grey: unclustered cloud.

Then, not surprisingly, there are sharp differences between the phylogenetic clusters in terms of number of taxonomy hits (ANOVA P-value below 0.0001, see Fig. 3). Cluster A proteins have usually a low number of hits, up to a few hundreds. Cluster C includes proteins with between 50 and 100 hits. Cluster D has a very low number of hits, below 50. Cluster B includes only proteins with more than 1000 taxonomy hits. The unclustered “cloud” contains proteins differing in hit number, from just a few up to more than one thousand.

**Figure 4:**
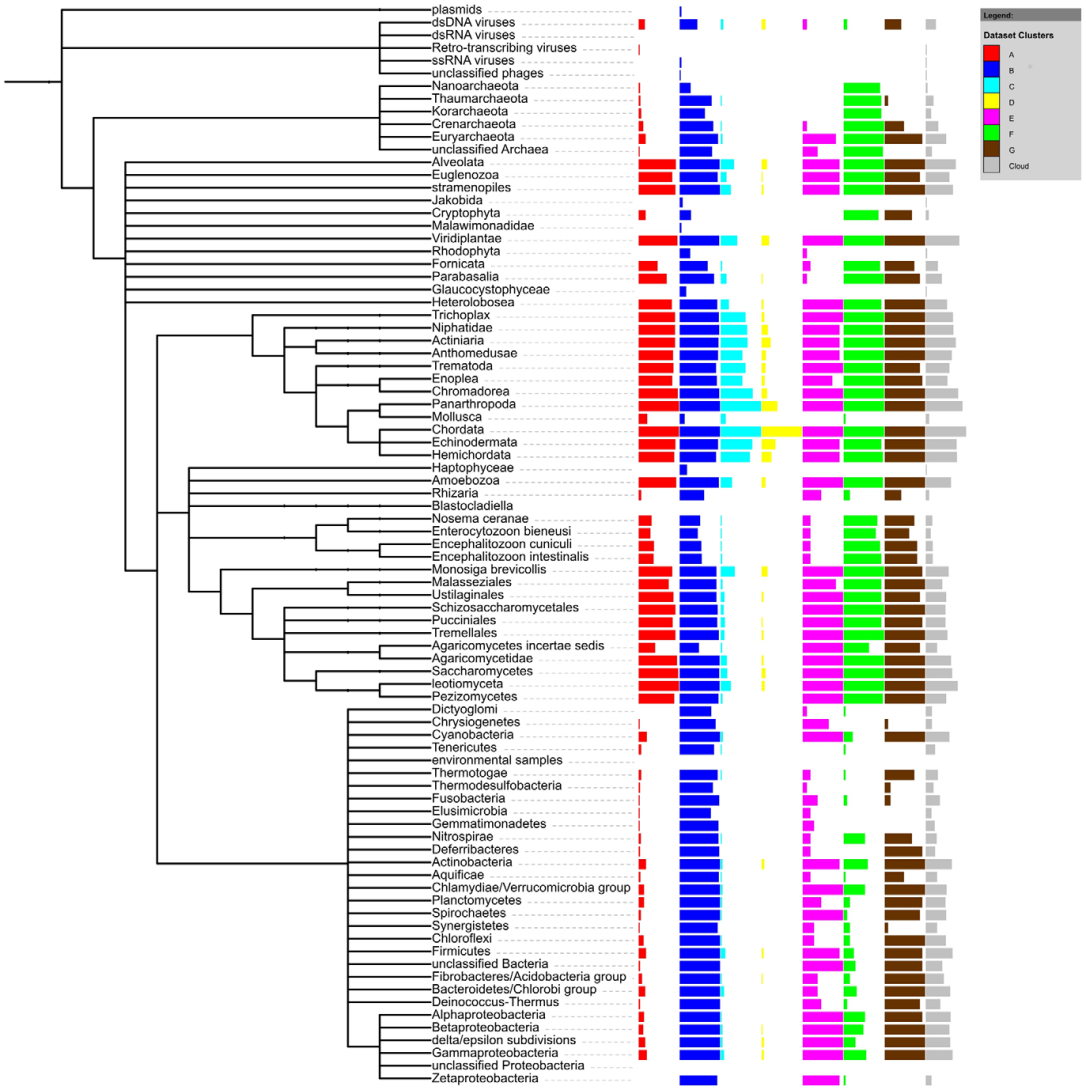
Counts of erythrocyte proteins in phylogenetic clusters mapped onto taxonomic classification. The classification has been shown up to a division/phylum level, except for Metazoa which have been expanded to show differences between different classes of animals (expanded to the equal level of depth) and Proteobacteria which have been expanded to include its classes. Red: cluster A, dark blue: cluster B, light blue: cluster C, yellow: cluster D, magenta: cluster E, green: cluster F, brown: cluster G, grey: unclustered cloud. Counts are normalized against cluster size.

The clusters differ not only in the number of taxonomy hits but also, quite expectedly, in their phylogenetic spread (see Fig. 4 and Fig. S1). Most widespread are the cluster B proteins, including more than 14% of the erythrocyte proteome, that are present in all eukaryotes, but also in all major bacterial groups, in Archaea and in dsDNA viruses. Second in line are cluster G proteins, just 1% of the RBC proteome, present in most taxa, but almost absent from three out of five archaeal taxa (Nanoarcheota, Thaumarcheota and Koracheota), also almost absent from 8 out of 23 bacterial taxa (e.g. Dictyoglomi, Tenericutes, Elusimicrobia, Gemmatimonadetes). Cluster F proteins, 2% of the RBC proteome, are present in all Archaea and most Eukaryota (excluding Rhodophyta and Jakobida), but absent from most bacterial taxa (modest presence in Proteobacteria, Chlamydiae, Actinobacteria and Nitrospirae). Cluster E (1%) is present throughout Eukaryotes, in Crenarcheota, and Eurarcheota, and has a patchy distribution in Bacteria (similar to cluster F, with addition of Cyanobacteria and Chrysiogenetes). Cluster A (as much as 38% of the RBC proteome) seems to be Eukaryote-specific, but with minor presence in bacterial and archaeal taxons (e.g. Proteobacteria, Cyanobacteria, Chloroflexi, Firmicutes, Actinobacteria, Eurarcheota). Cluster C (13% of the RBC proteome) appears to be Metazoa-specific, but with minor presence in plants, Amoebae, fungi, a few unicellular eukaryotic taxa, and in bacterial and archaeal taxons (e.g. Proteobacteria, Cyanobacteria, Eurarcheota). Cluster D (6%) is by large vertebrate-specific, yet with some presence in echinoderms, hemichordates, arthropods and plants.

The limitations of our clustering approach are reflected in the presence of the unclustered “cloud” including 26% of the proteome. An interesting separation is visible in the classification of viruses. HRBC proteins from all clusters have a significant number of homologs in genomes of dsDNA viruses (especially clusters B and G, for which approx. half of cluster members have homologues in this virus group) but almost none in dsRNA, ssRNA and retro-transcribing viruses.

To assess the depth of our approach, we attempted to reproduce the above analysis using orthologous groups instead of PSI-BLAST searches as a basis for protein clustering. However, not only a very small number of proteins could be mapped to orthologous groups (in total we have obtained 368 groups from the initial set of 1410 proteins), but also phylogenetic depth of absolute majority of them, as reported by eggNOG 2.0 [18] database, was very shallow. Only four groups had more than 10 species reported. Identification of an orthologous gene relies on the availability of complete genomes (630 are available in the eggNOG 2.0 database), which limits the depth of the search compared to using all proteins from a database such as RefSeq.

### Functional analysis of the clusters

**Figure 5:**
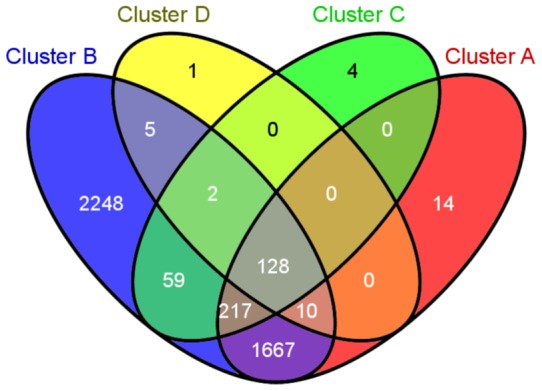
Venn diagram of species counts for the four largest (in terms of the number of HRBC proteins) clusters.

The species hit by proteins from each cluster overlap to different extents (see Fig. 5). Comparing four largest clusters in terms of number of proteins there are 2248 species specific to cluster B – these are mostly bacteria. There is also a huge number (1667) of species common to two major clusters, A and B. Also these are mostly bacterial species. There are 128 species common to four major clusters, and these include mostly animals, but also a few plants and fungi.

The proteins in the seven clusters are enriched in different functional annotations. In the gene set enrichment approach (see Methods), the method itself treats the conceptually different biological process annotations and molecular function annotations in the same manner. As expected, judging from the broad taxonomic spread, cluster B that is composed of proteins with very broad phylogenetic spread is involved in basic cellular functions while the other clusters, more focused in taxonomic spread, possess functions typical of multicellular organisms (see Table S1).

The cluster B proteins, 14% of the erythrocyte proteome, are present in all taxons, reflecting the most basic cellular machinery necessary for most living cells, e.g. processes such as glycolysis and pyruvate metabolism, molecular functions such as oxidoreductase activity, nucleotide binding (see Table S1). These proteins have sequence hits to more than 1000 species each. The cluster C may be linked to multicellularity, and accordingly is enriched in molecular functions involving membrane proteins, and processes such as cell adhesion. The vertebrate-specific cluster D is most likely made of proteins responsible for specific functions that evolved within this group. It includes secreted proteins, EF-hand calcium binding proteins (including the S100 family) and apoptotic proteins (albeit the significance of the latter is seen only before the multiple test correction). The eukaryote-specific cluster A is enriched in proteins involved in biological processes such as protein transport, ubiquitination pathway, and GTPase signalling. The relatively small and relatively ubiquitous clusters E, F and G are enriched in glutathione S-transferase domains, proteasome subunits, and WD40 repeats, respectively. No significant enrichment of functional terms is seen for the unclustered cloud.

Some more specific signalling pathways can be detected to be enriched within the clusters. The cluster C (multicellular eukaryotes: Metazoa, plants) is involved in cell movement, cell growth, cell development, and its specific pathways include the JAK/STAT signalling (e.g. STAT5B, STAT6 molecules) and the erbB pathway. Cluster A (eukaryote-specific) includes small GTPase signalling mediators, e.g. KRAS, RHOB, MAP2K1, MAP2K2 and MAPK1. Finally, the cluster D, with the narrowest species spectrum (present only in vertebrates), includes apoptosis signalling mediators such as TNF, TRADD, MAP3K1 and BMF. Functions of the proteins in the unclustered “cloud” do not reach high statistical significance.

**Figure 6:**
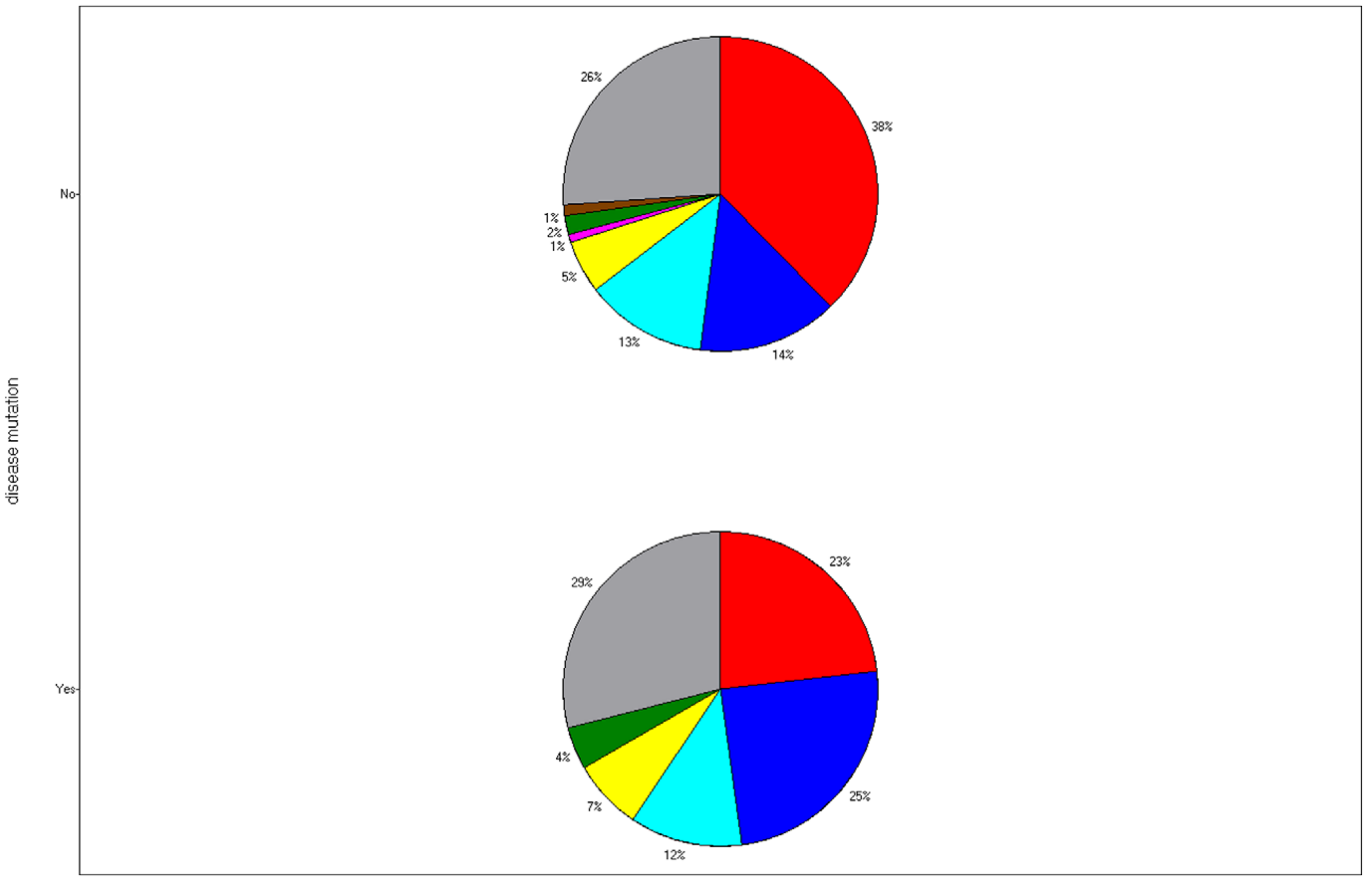
Fractions of proteins from different clusters among disease mutation proteins (lower pie chart) and other proteins (upper pie-chart). Red: cluster A, dark blue: cluster B, light blue: cluster C, yellow: cluster D, magenta: cluster E, green: cluster F, brown: cluster G, grey: unclustered cloud.

Another difference between the clusters can be gleaned by analysing disease-causing mutations, as included in the HUMSAVAR list in the Uniprot database, www.uniprot.org. Mutations are not evenly distributed in the erythrocyte clusters (See Fig. 6). The erythrocyte proteome studied herein includes 54 proteins with “disease mutations.” Cluster A (eukaryote-specific), which makes up 38% of the erythrocyte proteome, and includes 23% of erythrocyte disease mutation proteins, has significantly fewer mutations than expected by chance (binomial test p-value 0.007). Cluster B (ubiquitous), constituting 14% of the proteome, has 25% of disease mutation proteins, significantly more than expected by chance (p-value 0.006). Thus, the modest mutation sample available suggests that disease mutations are under-represented in the cluster specific to eukaryotic organisms while they tend to be overrepresented in the most common cluster that includes archaeal, bacterial and eukaryotic proteins.

## Discussion

In this study, we separated the red blood cell proteins into biologically relevant clusters using a visual approach to clustering by phylogenetic similarity. We obtained seven clear-cut clusters and a “cloud” of proteins whose phylogenetic profiles are specific. The clusters do differ in their phylogenetic profiles. Some clusters are taxonomically very widespread (cluster B) while others are specific to a taxonomic branch (e.g. the vertebrate-specific cluster D, Metazoa-specific cluster C). So, erythrocyte protein clusters are somehow “old” or “young” depending on their taxonomic distribution.

The clusters are over-represented with specific functions. Again, some functions are “older” and some seem to be acquired later in evolution. The only clusters that may be in part related to red blood cell formation are the vertebrate cluster D and the metazoan cluster C.

The most significant functional annotations elucidated in this work differ from those of d'Alessandro and colleagues [9] since they performed an analysis involving the totality of the erythrocyte proteome against the full human proteome while we analysed separate clusters, defined phylogenetically, against the erythrocyte proteome background. Nevertheless, some of the top functional categories found in this work overlap with those of d'Alessandro since the analysed protein sets were overlapping, and the software tool was conceptually similar (Ingenuity IPA and David). For individual protein clusters, we did not observe prevalence of annotations from the conceptually different general annotation types, i.e. biological processes and of molecular function. Both biological processes and molecular function terms occurred for most clusters (see Table S1). This probably results from the difficulty of strict separation of the two annotation types, i.e. specific molecular annotations are often linked to specific processes.

The different clusters might be expected to reflect three evolutionary steps important for the evolution of erythrocyte: 1) formation of the eukaryotic cell, 2) appearance of blood and erythrocytes, 3) loss of nuclei in mammalian erythrocytes. However, our phylogenetic profile clustering method too coarse-grained to capture exactly that level of detail. The chimeric origin of eukaryotes is not captured precisely by the clustering. Yet, it is to some degree reflected in the cluster B with the largest taxonomic spectrum (Bacteria, Archaea, Eukaryotes), where proteins do have both information-processing and metabolic (operational) functions, as expected for archaeal and bacterial origins, respectively [25]. Cluster F, which contains eukaryotic and archaeal proteins represents the known evolutionary history of proteasome. Cluster A, which contains mostly eukaryotic proteins, could correspond to establishing major metabolic constituents of a eukaryotic cell. Cluster C, having a narrower spectrum of higher eukaryotic species, mostly Metazoan, could correspond to the step of establishing tissues. And finally, cluster D, almost exclusive to Chordata could be linked functionally to erythropoiesis and occurrence of blood. The clusters identified do not allow to trace recent evolution of erythrocytes (loss of nuclei in mammals), probably because the starting point of the analysis was erythrocyte itself, i.e. an already “truncated” version of the cell (no nucleus).

The 28% of erythrocyte proteins are unclustered in our approach. This is a substantial part of the proteome. An example of these unclustered proteins are hemoglobins. The ancestral gene for hemoglobin is ancient and its products served many different roles in the history, freed from evolutionary pressure by gene duplication and whole genome duplication. Thus, hemoglobins, and many other proteins in the unclustered cloud represent a set of genes with highly complex history. Their separation (presence outside of any cluster) indicates that there is very little in common between these proteins. They might be an interesting set of proteins to analyze individually.

The cluster A has significantly fewer than expected disease-linked mutations in its genes, which is logical considering the functions crucial for eukaryotic life. Conversely, the most widespread cluster B including proteins common to all forms of life has over-representation of disease mutations, consistent with its functions involving basic cell metabolism.

Construction of phylogenetic profiles based on RefSeq database leads to a significant noise in the profile. For example, we have found that *E. coli* proteins were spread across 60 different taxonomy ids. At first, it seems that our approach is not reliable enough, compared to careful construction of phylomes. However, as we show in this study, the graph layout approach employed using CLANS software provides enough granularity to efficiently cluster proteins of a single cell, based on presence of homologous proteins across proteomes. Clustering based on orthology is not powerful enough because it relies on the availability of fully sequenced genomes and these data always lag behind draft genomes or partially sequenced organisms. On the other hand, our method is only efficient for coarse-grained evolutionary steps description. As clusters are perfectly separated, relations between clusters (other than distances) are missing, therefore relatively recent or very old changes, captured perfectly by other methods, might be missing from our analysis. As such, our approach might be a complement to more fine-grained methods, for which clustering based on phylogenetic profiles could provide a noise-resistant starting point.

## Supporting Information

Figure S1 **Counts of erythrocyte proteins in phylogenetic clusters mapped onto taxonomic classification.** The classification has been shown up to a “division/phylum” level, except for Metazoa which have been expanded to show differences between different classes of animals (expanded to the equal level of depth) and Proteobacteria which have been expanded to include its classes. Red: cluster A, dark blue: cluster B, light blue: cluster C, yellow: cluster D, magenta: cluster E, green: cluster F, brown: cluster G, grey: unclustered “cloud”.
(TIF)

Table S1 **Selected functional annotations overrepresented in the clusters, as calculated by the David tool** [**24**] **.** Annotations separated into Biological processes, including pathways (upper part of the table) and Molecular functions, including subcellular localisation (lower part of the table). Percent: percentage of proteins within a cluster annotated with a term. Fold enrichment: enrichment in the functional annotation within the cluster as compared to the background set (whole RBC proteome). Benjamini: enrichment P-value with Benjamini correction for multiple testing. Annotation category: the database from which given annotation cam.
(DOC)
