# Supplementary figures and images for: Distinct Protein Classes in Human Red Cell Proteome Revealed by Similarity of Phylogenetic Profiles
Source: PLoS One. 2013 Jan 21;8(1):e54471. doi: 10.1371/journal.pone.0054471 (PMC3549994; doi:10.1371/journal.pone.0054471)

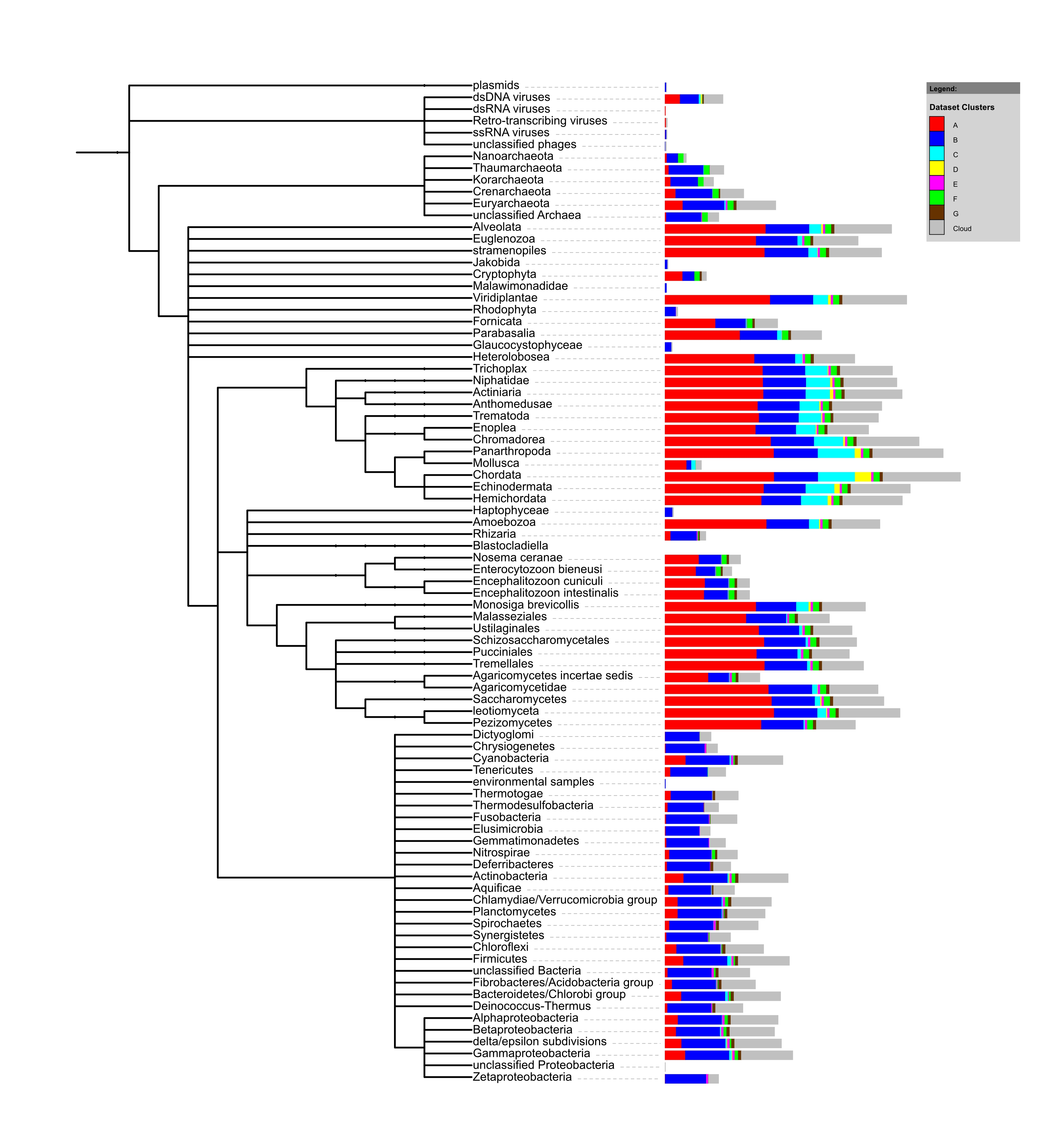

Supplement: Figure S1 — Counts of erythrocyte proteins in phylogenetic clusters mapped onto taxonomic classification. The classification has been shown up to a “division/phylum” level, except for Metazoa which have been expanded to show differences between different classes of animals (expanded to the equal level of depth) and Proteobacteria which have been expanded to include its classes. Red: cluster A, dark blue: cluster B, light blue: cluster C, yellow: cluster D, magenta: cluster E, green: cluster F, brown: cluster G, grey: unclustered “cloud”. (TIF) [file pone.0054471.s001.tif]
