# Supplementary material for: Distinct Protein Classes in Human Red Cell Proteome Revealed by Similarity of Phylogenetic Profiles
Source: PLoS One. 2013 Jan 21;8(1):e54471. doi: 10.1371/journal.pone.0054471 (PMC3549994; doi:10.1371/journal.pone.0054471)
Supplement: Table S1 — Selected functional annotations overrepresented in the clusters, as calculated by the David tool [24] . Annotations separated into Biological processes, including pathways (upper part of the table) and Molecular functions, including subcellular localisation (lower part of the table). Percent: percentage of proteins within a cluster annotated with a term. Fold enrichment: enrichment in the functional annotation within the cluster as compared to the background set (whole RBC proteome). Benjamini: enrichment P-value with Benjamini correction for multiple testing. Annotation category: the database from which given annotation cam. (DOC) [file pone.0054471.s002.doc]

**Supplementary Table 1.**

Selected functional annotations overrepresented in the clusters, as calculated by the David tool [24]. Annotations separated into Biological processes, including pathways (upper part of the table) and Molecular functions, including subcellular localisation (lower part of the table).

Percent: percentage of proteins within a cluster annotated with a term. Fold enrichment: enrichment in the functional annotation within the cluster as compared to the background set (whole RBC proteome). Benjamini: enrichment P-value with Benjamini correction for multiple testing. Annotation category: the database from which given annotation came.

| Cluster | Annotation category | Term | percent | P-value | Fold Enrich­ment | Benjamini |
| --- | --- | --- | --- | --- | --- | --- |
| **Biological processes, including pathways** | | | | | | |
|  |  |  |  |  |  |  |
| A | KEGG_PATHWAY | hsa04140:Regulation of autophagy | 1.86 | 0.0019 | 2.84 | 0.04 |
| A | KEGG_PATHWAY | hsa04144:Endocytosis | 5.99 | 8.03E-004 | 1.68 | 0.03 |
| A | GOTERM_BP_FAT | GO:0007264~small GTPase mediated signal transduction | 8.06 | 2.08E-004 | 1.62 | 0.03 |
| A | GOTERM_BP_FAT | GO:0006511~ubiquitin-dependent protein catabolic process | 8.47 | 0.01 | 1.37 | 0.46 |
| A | KEGG_PATHWAY | hsa04120:Ubiquitin mediated proteolysis | 4.96 | 5.83E-006 | 2.2 | 3.21E-004 |
| A | GOTERM_BP_FAT | GO:0015031~protein transport | 22.73 | 1.92E-017 | 1.82 | 3.25E-014 |
|  |  |  |  |  |  |  |
| B | KEGG_PATHWAY | hsa00230:Purine metabolism | 9.44 | 8.34E-06 | 3.01 | 2.25E-04 |
| B | KEGG_PATHWAY | hsa00020:Citrate cycle (TCA cycle) | 3.89 | 0.0014 | 4.19 | 0.028 |
| B | GOTERM_BP_FAT | GO:0006732~coenzyme metabolic process | 1.10.2000 | 2.56E-006 | 3.36 | 1.87E-04 |
| B | GOTERM_BP_FAT | GO:0051186~cofactor metabolic process | 12.22 | 2.21E-008 | 3.62 | 2.43E-06 |
| B | GOTERM_BP_FAT | GO:0006457~protein folding | 11.67 | 2.34E-007 | 3.38 | 1.83E-05 |
| B | KEGG_PATHWAY | hsa00620:Pyruvate metabolism | 6.67 | 4.37E-006 | 4.1 | 1.77E-04 |
| B | SP_PIR_KEYWORDS | glycolysis | 6.11 | 1.33E-007 | 6.43 | 6.13E-06 |
| B | GOTERM_BP_FAT | GO:0006096~glycolysis | 7.22 | 3.80E-009 | 6.42 | 6.95E-07 |
| B | GOTERM_BP_FAT | GO:0044275~cellular carbohydrate catabolic process | 8.89 | 3.20E-009 | 5.27 | 7.02E-07 |
| B | KEGG_PATHWAY | hsa00010:Glycolysis / Gluconeogenesis | 8.89 | 2.65E-009 | 4.5 | 2.15E-07 |
| B | GOTERM_BP_FAT | GO:0016052~carbohydrate catabolic process | 9.44 | 5.82E-010 | 5.34 | 1.60E-07 |
| B | GOTERM_BP_FAT | GO:0019318~hexose metabolic process | 12.78 | 2.27E-010 | 4.19 | 8.29E-08 |
| B | GOTERM_BP_FAT | GO:0005996~monosaccharide metabolic process | 13.89 | 4.53E-011 | 4.12 | 2.49E-08 |
| B | GOTERM_BP_FAT | GO:0055114~oxidation reduction | 18.89 | 7.34E-016 | 4.27 | 8.5E-013 |
|  |  |  |  |  |  |  |
| C | KEGG_PATHWAY | hsa04012:ErbB signaling pathway | 4.51 | 0.0027 | 4.29 | 0.08 |
| C | KEGG_PATHWAY | hsa04630:Jak-STAT signaling pathway | 3.87 | 5.26E-04 | 6.90 | 0.02 |
| C | KEGG_PATHWAY | hsa05220:Chronic myeloid leukemia | 5.16 | 3.79E-04 | 4.91 | 0.034 |
| C | GOTERM_BP_FAT | GO:0007155~cell adhesion | 8.39 | 0 | 2.76 | 0.44 |
|  |  |  |  |  |  |  |
| D | GOTERM_BP_FAT | GO:0042981~regulation of apoptosis | 24.24 | 1.58E-004 | 2.87 | 0.17 |
|  |  |  |  |  |  |  |
| E | No significant biological process terms for this cluster | | | | | |
|  |  |  |  |  |  |  |
| F | KEGG_PATHWAY | hsa03050:Proteasome | 51.8 | 3.4E-013 | 11.8 | 1.7E-12 |
|  |  |  |  |  |  |  |
| G | No significant biological process terms for this cluster | | | | | |
|  |  |  |  |  |  |  |
| **Molecular functions, including subcellular localisation** | | | | | | |
|  |  |  |  |  |  |  |
| A | INTERPRO | IPR001806:Ras GTPase | 5.37 | 1.50E-006 | 2.17 | 0 |
| A | INTERPRO | IPR013753:Ras | 5.58 | 2.00E-007 | 2.26 | 0 |
|  |  |  |  |  |  |  |
| B | GOTERM_CC_FAT | GO:0005739~mitochondrion | 16.11 | 1.72E-007 | 2.7 | 2.98E-05 |
| B | GOTERM_MF_FAT | GO:0005524~ATP binding | 34.44 | 2.55E-008 | 1.87 | 1.87E-06 |
| B | GOTERM_MF_FAT | GO:0030554~adenyl nucleotide binding | 36.11 | 4.54E-009 | 1.89 | 9.99E-007 |
| B | GOTERM_MF_FAT | GO:0001882~nucleoside binding | 37.22 | 2.93E-009 | 1.88 | 1.29E-06 |
| B | SP_PIR_KEYWORDS | oxidoreductase | 17.78 | 8.70E-017 | 4.78 | 3.1E-014 |
|  |  |  |  |  |  |  |
| C | GOTERM_CC_FAT | GO:0016021~integral to membrane | 21.29 | 5.21E-004 | 1.76 | 0.04 |
| C | SP_PIR_KEYWORDS | transmembrane | 18.71 | 8.18E-006 | 2.33 | 0 |
| C | GOTERM_CC_FAT | GO:0005886~plasma membrane | 37.42 | 2.15E-006 | 1.68 | 5.22E-04 |
|  |  |  |  |  |  |  |
| D | GOTERM_CC_FAT | GO:0005576~extracellular region | 18.18 | 2.08E-004 | 3.57 | 0.04 |
| D | GOTERM_MF_FAT | GO:0005509~calcium ion binding | 16.67 | 0 | 3.27 | 0.2 |
| D | PFAM | PF01023:S_100 | 10.61 | 3.31E-007 | 17.11 | 5.73E-05 |
|  |  |  |  |  |  |  |
| E | PFAM | PF00043:GST_C | 44.44 | 4.49E-007 | 154 | 6.29E-06 |
| E | INTERPRO | IPR010987:Glutathione S-transferase. C-terminal-like | 55.56 | 1.04E-008 | 115.46 | 1.15E-07 |
|  |  |  |  |  |  |  |
| F | GOTERM_CC_FAT | GO:0005839~proteasome core complex | 51.85 | 5.54E-020 | 32.02 | 2.8E-018 |
| F | GOTERM_MF_FAT | GO:0004298~threonine-type endopeptidase activity | 51.85 | 9.90E-021 | 35.91 | 6.6E-019 |
| F | PFAM | PF00227:Proteasome | 51.85 | 2.48E-021 | 39.93 | 1.0E-019 |
| F | SP_PIR_KEYWORDS | threonine protease | 51.85 | 1.80E-021 | 40.93 | 8.8E-020 |
|  |  |  |  |  |  |  |
| G | PFAM | PF00400:WD40 | 80 | 1.05E-018 | 44.8 | 2.7E-017 |
| G | INTERPRO | IPR001680:WD40 repeat | 93.33 | 1.30E-023 | 48.49 | 2.9E-022 |
